# Microbiome and lipidomic analysis reveal the interplay between skin bacteria and lipids in a cohort study

**DOI:** 10.3389/fmicb.2024.1383656

**Published:** 2024-04-11

**Authors:** Min Li, Evguenia Kopylova, Junhong Mao, Jin Namkoong, Jon Sanders, Joanna Wu

**Affiliations:** ^1^Colgate-Palmolive Company, Global Technology Center, Piscataway, NJ, United States; ^2^Clarity Genomics, San Diego, CA, United States

**Keywords:** skin microbiome, skin lipids, lipidomics, microbiome–lipid interaction, 16S rRNA sequencing

## Abstract

Human skin acts as a protective barrier between the body and the external environment. Skin microbiome and intercellular lipids in the stratum corneum (SC) are essential for maintaining skin barrier function. However, the interplay between skin bacteria and the lipids is not fully understood. In this study, we characterized the skin microbiome and SC lipid profiles from the forearm and face in a cohort of 57 healthy participants. 16S rRNA gene sequencing showed the skin microbial composition is significantly different between body locations and genders. Female forearm samples have the highest microbial diversity. The relative abundance of *Staphylococcus hominis, Micrococcus luteus, Corynebacterium tuberculostearicum, Finegoldia magna,* and *Moraxellaceae* sp. are significantly higher in the forearm than the face. The predictive functional analysis of 16S rRNA gene sequencing by Phylogenetic Investigation of Communities by Reconstruction of Unobserved States (PICRUSt2) and ANCOM-BC showed different bacterial metabolic pathway profiles between body locations or genders, and identified 271 differential pathways, including arginine and polyamine biosynthesis, chorismate biosynthesis pathways, which are more abundant in the female forearm, and sulfur oxidation pathway, which is more abundant in the male face. The SC lipid profiles differ between the body locations as well. Total free fatty acids (FFA), cholesterol sulfate and sphingosine are more abundant in the face. Dihydro-/6-hydroxy/phyto-ceramides are more abundant in the forearm. The correlation analysis of 16S rRNA gene sequencing and lipids revealed novel interplay between the bacteria and skin lipids. Shannon entropy and *S. hominis* negatively correlated with FFA, cholesterol sulfate and sphingosine; while positively correlated with dihydro-/6-hydroxy/phyto-ceramides. The correlation of predictive pathway profiles and lipids identified pathways involved in amino acids metabolism, carbohydrates degradation, aromatic compounds metabolism and fatty acid degradation metabolism are positively correlated with dihydro-/6-hydroxy/phyto-ceramides and negatively correlated with FFA, cholesterol sulfate and sphingosine. This study provides insights on the potential correlation between skin microbiome and lipids.

## Introduction

The skin is the largest organ of the human body and acts as a protective barrier between the body and the external environment. The intercellular lipids in the outermost layer of the skin, the stratum corneum (SC), are one of the fundamental components to maintain the skin barrier function (Knox and O’Boyle, 2021). These lipids suppress excessive water and electrolyte loss and prevent the compounds from the environment permeating into the epidermis and the dermis, and thereby provoke an immune response (van Smeden and Bouwstra, 2016). The composition of the skin lipid matrix is dominated by three classes: ceramides, cholesterol, and free fatty acids (FFAs) (Knox and O’Boyle, 2021). Ceramides are the most common constituent, accounting for 40–50% of the total intercellular lipids (Choi and Maibach, 2005). Depending on the type of sphingosine and the type of fatty acid bound together, there are 12 different subclasses of ceramides identified in human SC (Murphy et al, 2022). Among these ceramides subclasses, ceramide esterified omega fatty acids (EOS), phytoceramide saturated fatty acids (NP), phytoceramide alpha-hydroxy fatty acids (AP), also called ceramides 1, 3, and 6-II respectively, are considered essential ceramides that excel in supporting skin health by preserving the integrity of the lamellar layer (Coderch et al., 2003; Huey-Chun and Chang, 2008). They are also widely used in a variety of skincare products. The distribution and composition of the skin lipids vary across different body locations (Starr et al., 2016), and are influenced by age, gender and seasonal variations (Rogers et al., 1996; Starr et al., 2016; Choe et al., 2018). Studies have demonstrated that alterations in the SC lipid composition can lead to impaired skin barrier functions, giving rise to skin disorders such as psoriasis and atopic dermatitis (Pietrzak et al., 2010; Emmert et al., 2021). Therefore, understanding the composition of skin lipids profile and the impact of host and external factors on it is critical for skin health.

The SC is also colonized by a variety of living microorganisms, called the skin microbiome, which is essential for maintaining skin barrier function. The microbes form an invisible ecosystem that protects the skin from opportunistic pathogens, contributes to the production of essential nutrients and educates the immune system to ensure human health (Grice, 2015; Byrd et al., 2018). The skin microbial composition highly depends on the topographic locations of the human body and varies by age and gender (Grice et al., 2008). The skin barrier and microbiome have a symbiotic relationship, influencing one another through physical, chemical, and immunological interactions. The skin microbiome can secrete the components that make up the lipid structure. For instance, *Staphylococcus epidermidis* produces a sphingomyelinase that acquires essential nutrients for the bacteria and indirectly assists the host in producing ceramides to help build the skin lipids (Zheng et al., 2022). Meanwhile, epidermal lipids can serve as a nutrient source for the skin microbiome (Tchoupa et al., 2023). Pathogenic microorganisms are also directly inhibited by some lipids. For example, sapienic acid from the SC can effectively inhibit pathogenic *S. aureus* (Moran et al., 2017). Therefore, the cross-talk between the skin microbiome and lipids is very important to maintain skin barrier function, however, these interactions are not fully understood.

In this study, we characterized the skin microbiome and SC lipids profiles from the forearm and face in a cohort of 57 healthy participants using 16S rRNA gene sequencing and lipidomic analysis. The objective was to understand the impact of host factors such as age, gender, skin type and body location on skin microbiome and lipid profile, and to explore the interaction of skin bacteria and lipids. We also explored the mechanism of the relationship by using predictive functional analysis.

## Materials and methods

### Study design and sample collection

**Figure 1:**
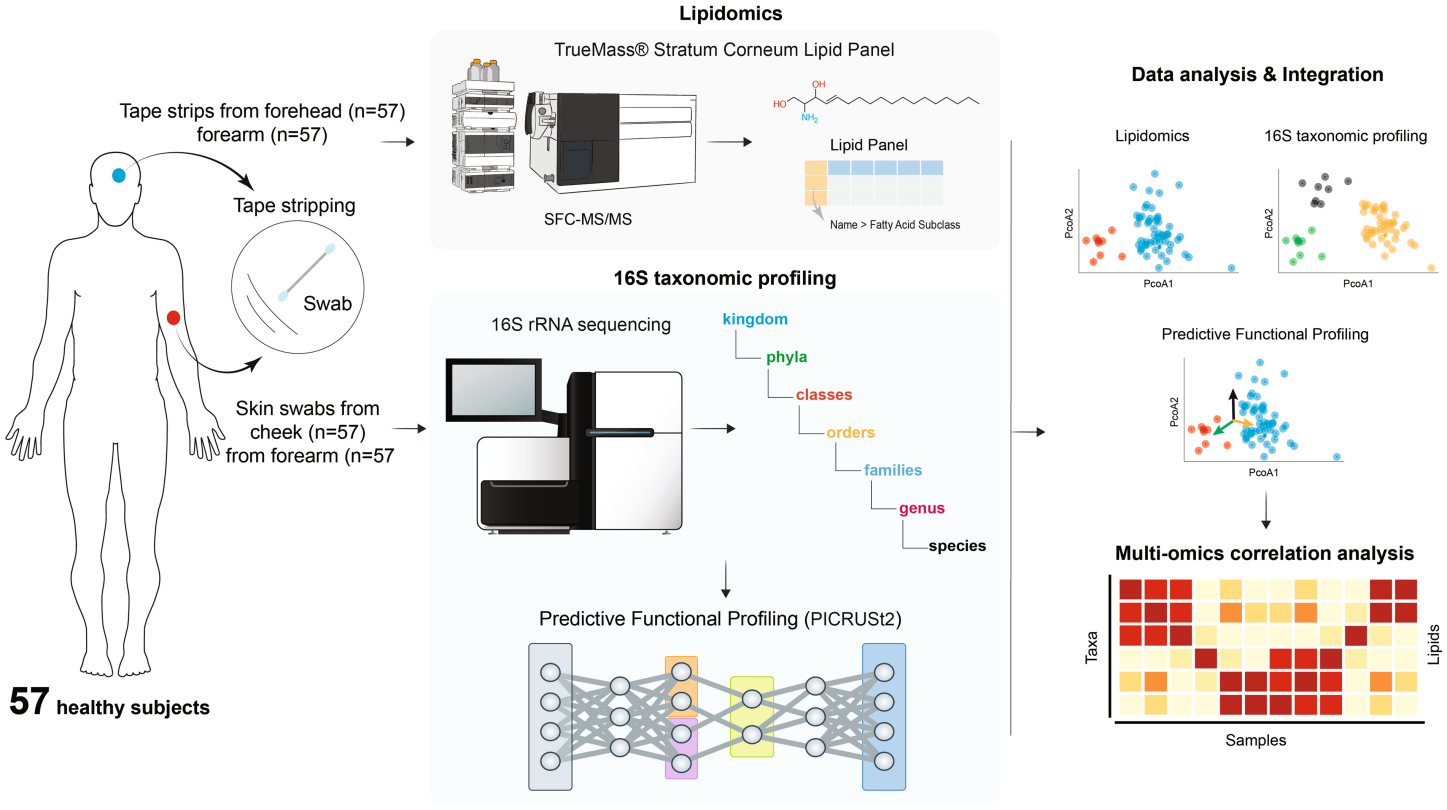
Overview of the study design.

The study was approved by Institutional review board of Concordia Clinical Research, Inc. (IBR Committee No. 188Z) (Cedar knolls, NJ, USA). A total of 57 healthy participants from Piscataway, New Jersey, USA were recruited. The study design was illustrated in Figure 1 (Gomes et al., 2023). The informed consent was signed by each participant. Prior to sampling, all participants were provided with a questionnaire in which they were asked for age, gender and skin type (oily, dry, normal). Participants were instructed not to take a shower or wash their face on the morning of the sample collection day. They were also instructed not to apply any products on the face and both forearms, including but not limited to soaps, shower gels, lotions, creams, oils, sunscreen and makeup.

One skin swab was collected from one forearm and cheek using a swabbing technique for microbiome analysis. A 5x5cm area of the skin was sampled by swabbing the skin for 30 s with a sterile flock swab, which was dipped into an aliquot of phosphate buffered saline (PBS). The lateral edge of the swab was rubbed across the entire defined area while being rotated between the thumb and forefinger for 30 s. More specifically, the rotating swab was rubbed back and forth in a cross-wise manner in the defined area in the same fashion for each participant to maintain consistency. After 30 s, the head of each swab was placed into a sterile microcentrifuge tube and aseptically cut from the breakpoint of the handle before closing the tube lid. All the samples were frozen at −80°C until further analysis.

Tape stripping was done on the surface of one forearm and forehead using D-Squame standard sampling disc, 22 mm in diameter (Clinical & Derm, LLC, Dallas, Texas) for lipidomic analysis. The tape was applied to the skin surface and briefly pressed with a standardized pressure pen of 225 g/cm2 (D-Squame pressure instrument D500, Clinical & Derm, LLC). On the same spot, a total of 4 consecutive tapes were collected. Each collected tape was placed in a storage card (D-Squame standard storage card D120, Clinical & Derm, LLC) and stored in −80°C until the analysis.

### 16S rRNA gene sequencing

V1-3 hypervariable region of 16S rRNA gene sequencing was conducted by RTL Genomics (Lubbock, Texas) as previously described (Li et al., 2023). DNA was extracted via KingFisher FLEX instrument (ThermoFisher Scientific, Inc., Waltham, Massachusetts) and using Zymo ZR-96 magbead kit (Zymo Research, Irvine, California) following manufacturer’s instructions. The extraction protocol was modified to include a mechanical lysis step with a Qiagen TissueLyser. V1-3 region of 16S rRNA gene was amplified for sequencing in two-step, independent reactions using HotStar Taq Master Mix Kit (Qiagen) with 28F-519R primers (28F: 5”GAG TTT GAT CNT GGC TCA G 3″; 519R: 5” GTN TTA CNG CGG CKG CTG 3″). PCR amplification included 0.5 μl of 5 μM forward primer, 0.5 μl of 5 mM reverse primer, 5 μl of DNA template, and 14 μl of Taq Master Mix. To encourage amplification in low biomass samples, 2 μl BSA and 2 μl MgCl_2_ were added to reactions. The negative control was a reaction mixture with no template DNA. PCR reaction conditions included initial denaturation at 95°C for 5 min, then 10 cycles of 94°C for 30 s, 50°C for 90 s (+0.5°C per cycle), 72°C for 1 min, followed by 25 cycles of 94°C for 30 s, 54°C for 90 s, 72°C for 1 min, and finally, one cycle of 72°C for 10 min and 4°C hold. Barcoding PCR reaction conditions included initial denaturation at 95°C for 5 min, then 10 cycles of 94°C for 30 s, 54°C for 40 s, 72°C for 1 min, followed by one cycle of 72°C for 10 min and 4°C hold. Amplification products were visualized with eGels (Life Technologies). Products were then pooled equimolar and each pool was size selected in two rounds using SPRIselect beads (BeckmanCoulter) in a 0.75 ratio for both rounds. Size selected pools were then quantified using Qubit 4 fluorometer (Life Technologies) and loaded on an Illumina MiSeq 2 × 300 flow cell at 10 pM for sequencing.

### Skin lipid analysis

Stratum corneum lipids were analyzed by Metabolon Inc. (Morrisville, North Carolina). Total free fatty acids, cholesterol, cholesterol sulfate and ceramides were measured by SFC-MS/MS using TrueMass® Stratum Corneum Lipid Panel (Emmert et al., 2021). Tissue samples were extracted with hexane after addition of a known amount of surrogate standard solution consisting of stable-labeled forms of ceramides, fatty acids, cholesterol and cholesterol sulfate. The organic extracts were combined and evaporated to dryness. The dried extract was reconstituted, and an aliquot was analyzed on a Waters UPC2/Sciex QTrap 5,500 mass spectrometer SFC-MS/MS system in MRM mode using characteristic parent-fragment mass transitions for each analyte trace. The quantitation of the individual lipid species was based on a single-point calibration using a surrogate standard. Concentrations were determined by peak area comparisons of the individual lipid species with the peak areas of their corresponding surrogate standards for which concentrations are known. Concentrations were given in pmol/tape for individual analytes as well as each lipid class. Additionally, the percentage composition of 10 individual ceramide subtypes is listed for each sample (Table 1), including ceramide alpha-hydroxy fatty acids (AS), ceramide esterified omega fatty acids (EOS), ceramide saturated fatty acids (NS), dihydroceramide alpha-hydroxy fatty acids (ADS), 6-hydroxyceramide alpha-hydroxy fatty acids (AH), phytoceramide alpha-hydroxy fatty acids (AP), 6-hydroxyceramide esterified omega fatty acids (EOH), dihydroceramide saturated fatty acids (NDS), 6-hydroxyceramide saturated fatty acids (NH), phytoceramide saturated fatty acids (NP).

**Table 1:** Ceramides subtypes measured by TrueMass® Stratum Corneum Lipid Panel in this study.

Ceramides class		Sphingosine base
Ceramides	Ceramide alpha-hydroxy fatty acids (AS)	Sphingosine
	Ceramide esterified omega fatty acids (EOS)	
	Ceramide saturated fatty acids (NS)	
Dihydroceramide	Alpha-hydroxy-dihydrosphingosine (ADS)	Dihydrosphingosine
	Non-hydroxy-dihydrosphingosine (NDS)	
6-Hydroxyceramide	6-hydroxyceramide alpha-hydroxy fatty acids (AH)	6-Hydroxysphingosine
	6-hydroxyceramide esterified omega fatty acids (EOH)	
	6-hydroxyceramide saturated fatty acids (NH)	
Phytoceramide	Phytoceramide alpha-hydroxy fatty acids (AP)	Phytosphingosine
	Phytoceramide saturated fatty acids (NP)	

### Data analysis

#### 16S rRNA gene sequencing data analysis

Raw FASTQ sequencing data (forward and reverse reads) was imported into QIIME2 (version 2022.2.0) (Bolyen et al., 2019). Quality control analysis identified lower quality regions in the first 20 nucleotides (primers) and, notably, in the last 20 nucleotides for the forward reads and the last 40 nucleotides for the reverse reads (as Phred read quality scores dropped below 20). To ensure sufficient overlap between the forward and reverse reads, the primers and the final 20 nucleotides were trimmed from both forward and reverse reads. The DADA2 (Callahan et al., 2016) plugin in QIIME2 was used to generate an ASV feature table with 6,301 ASVs (*qiime dada denoise-paired* command, with trimming of leading and trailing low quality nucleotides). A phylogenetic tree was built from the ASV sequencing using mafft (Katoh and Standley, 2013) and fasttree (Price et al., 2010) (*qiime phylogeny align-to-tree-mafft-fasttree* command). The resulting phylogenetic tree served for unweighted UniFrac (Lozupone and Knight, 2005) diversity metrics computation. Alpha diversity was explored as a function of sampling depth, and a rarefaction depth of 3,940 was selected for core diversity analyses because this was the highest sampling depth at which all 114 samples were retained, and Shannon alpha diversity appeared to level off after sampling depth of 2000 (*qiime diversity alpha-rarefaction* command). Alpha and beta diversity were computed using core diversity analysis (*qiime diversity core-metrics-phylogenetic*). Group significance analysis (Kruskal-Wallis for alpha diversity and PERMANOVA (Anderson, 2001) for beta diversity) was also computed (*qiime diversity alpha-group-significance* and *qiime beta-group-significance* commands). To assign taxonomy to the ASV sequences, we trained our own taxonomic classifier. First, the pre-formatted Silva 138 SSURef NR99 full-length sequences and taxonomy database (Quast et al., 2013; Robeson et al., 2021) were downloaded from QIIME2 data resources. Then, 550 nucleotides spanning the V1-3 hypervariable region of 16S rRNA gene were extracted from the full-length sequences (*qiime feature-classifier extract-reads* command with 28F-519R primers). A Naive Bayes classifier was trained on these extracted regions and the model then applied to classifying the ASVs (*qiime feature-classifier classify-sklearn* command). For species-specific analyses, the ASV feature table was collapsed to species level (*qiime taxa collapse* command) and converted to relative abundance (*qiime feature-table relative-frequency* command). Differential abundance analysis was run using ALDEx2 (v1.3.2) (Fernandes et al., 2014) with Benjamini–Hochberg correction and ANCOM-BC (Lin and Peddada, 2020) with Holm–Bonferroni correction using the species-level feature table. Differential abundance analysis for Shannon entropy was performed using pairwise Kruskal–Wallis with Benjamini–Hochberg correction.

#### Predictive functional analysis by phylogenetic investigation of communities by reconstruction of unobserved states (PICRUSt2)

The ASV feature table and sequences were input into the Phylogenetic Investigation of Communities by Reconstruction of Unobserved States (PICRUSt2) plugin in QIIME2 (*qiime picrust2 full-pipeline* command) (Janssen et al., 2018; Douglas et al., 2020) to generate predicted MetaCyc pathways. PICRUSt2 performs phylogenetic placement of the ASVs into a reference tree and then estimates the functional potential of the ASVs based on the known functions associated with the reference organisms in the tree. The default reference tree was used. Six of the 6,301 ASVs aligned poorly to the references and were excluded from downstream analysis. A total of 410 pathways were predicted. The Bray-Curtis distance metric was used for beta diversity analysis. Differential pathway abundance analysis was run using ANCOM-BC.

#### Lipids data analysis

The Euclidean distance metric was selected for beta diversity analysis due to the nature of the data, which consists of absolute concentration of lipids.

#### Integration and correlation analysis

**Figure 2:**
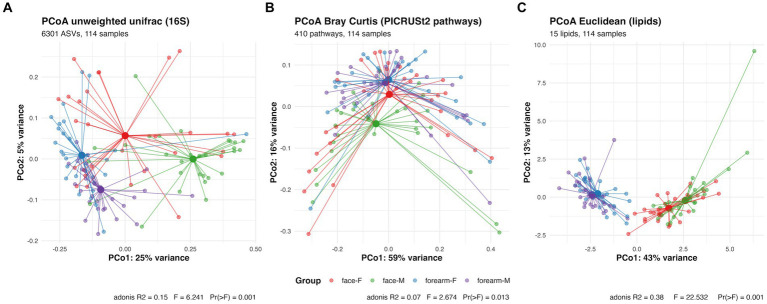
PCoA plot and PERMANOVA analysis of skin microbial composition characterized by **(A)** 16S rRNA gene sequencing **(B)** predictive metabolic pathway profiles analyzed by PICRUSt2 using 16S rRNA gene sequencing data **(C)** skin lipidomic profiles characterized by SFC-MS/MS. F, female; M, male.

Species-lipids, species-pathway and pathway-lipids correlation analysis was performed using Hierarchical All-against-All association testing HAllA (version 0.8.20) (Ghazi et al., 2022) using Spearman correlation and Benjamini–Hochberg false-discovery rate correction (*q*-value). Complementary to univariate analysis performed using ANCOM-BC and ALDEx2, supervised multivariate analysis using Random Forest was applied to gain insights into the predictive capabilities of the species microbiome and lipids with regard to location and gender group classification. The Boruta package (v.8.0.0) (Kursa and Rudnicki, 2010) was used for feature selection, with a maximum of 10,000 runs. Subsequently, a Random Forest model was constructed using the selected features, leading to an out-of-bag (OOB) error rate of 14.91% based on 1,000 trees, as depicted in Supplementary Figure S1. A multidimensional scaling (MDS) plot was generated using the proximity matrix of the Random Forest model (rescaled to the range of [−1, 1] to standardize the axes and facilitate a clearer visualization of the correlation between lipids, 16S and pathway variables and the ordination axes) and PICRUSt2 predicted pathways, together with Random Forest selected features driving the separation, were fitted on the MDS plot using the envfit function from the Vegan package, see Supplementary Figure S2A. The contribution of different species to selected PICRUSt2 predicted pathways is shown in Supplementary Figure S2B. The statistical method adonis2 from the Vegan R package (v.2.6-4) was used to assess the significance of variation in location and gender explained by 16S species (unweighted unifrac), PICRUSt2 predicted pathways (Bray–Curtis) and lipids (Euclidian), see Figure 2. Pairwise PERMANOVA analysis with Holm–Bonferroni correction was performed using the pairwise.adonis function (based on a loop using adonis2) (Arbizu, 2020).

## Results

### Skin microbial composition and predictive functional metagenomic profiles vary by body sites and gender

**Figure 3:**
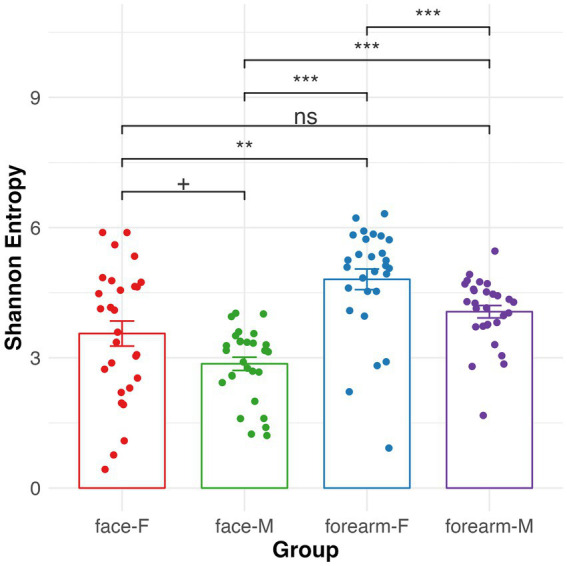
Alpha diversity of skin microbiome in different body sites and gender characterized by 16S rRNA gene sequencing. Kruskal–Wallis pairwise *q*-values represent ‘***’ 0–0.001, ‘**’ 0.001–0.01, ‘*’ 0.01–0.05, ‘+’ 0.05–0.1. F, female; M, male.

Alpha diversity (Shannon entropy) of the skin microbiome was significantly different between face and forearm samples, as well as between male and female samples (Figure 3). Female forearm samples have the highest microbial diversity, and male face samples have the lowest diversity. No significant change in alpha diversity was observed for skin type or age group (Supplementary Figure S3).

Beta diversity of the skin microbiome (PCoA analysis using unweighted unifrac distance) showed significant separation of the samples by body location (PC1, 25% explained variance) and gender (PC2, 5% explained variance) (Figure 2A). Pairwise PERMANOVA results showed *q*-value = 0.006 for all pairwise comparisons between location or gender. Discriminating bacteria between body location or gender are shown in Supplementary Figure S4. For instance, *Staphylococcus hominis* (*q*-value < 0.001)*, Corynebacterium tuberculostearicum* (*q*-value <0.001)*, Micrococcus luteus* (*q*-value = 0.003)*, Finegoldia magna* (*q*-value < 0.001) and *Moraxellaceae* sp. (*q*-value = 0.002) are more location specific. The relative abundance of these species were significantly higher in the forearm than the face. While *Streptococcus* sp. and *S. epidermidis* are more gender specific (ANCOM-BC location *q*-value >0.05 for both and gender *q*-value = 0.013 and *q*-value = 0.047, respectively). The relative abundance of *Streptococcus* sp. is higher in females than males, and *S. epidermidis* is higher in males than females especially in face samples.

To further investigate the functional potentials of the skin microbiome in this cohort, PICRUSt2 analysis was performed to predict MetaCyc metabolic pathways using 16S rRNA gene ASV data. In contrast to the skin microbial composition profiles, the PCoA plot for predicted functional profiles (Figure 2B) showed significant separation of the samples only for male face and forearm (*q*-value = 0.09) and male face and female forearm (*q*-value = 0.09). ANCOM-BC analysis identified 271 metabolic pathways (*q*-value <= 0.05) showing differential abundance between location or gender. The differential pathways are listed in Supplementary Table S1. To investigate the relationship between differentially abundant predicted pathways and the measured species and lipids, a Random Forest model was built on skin lipids and microbiome species data and a multidimensional scaling (MDS) plot generated using sample proximities (Supplementary Figures S1, S2). Differentially abundant predicted pathways were fitted onto the MDS plot and illustrated pathway distribution across sample groups. This analysis revealed stronger correlation of pathways to the forearm, especially in females, such as chorismate metabolism (ALL-CHORISMATE-PWY, *r*^2^ = 0.28), arginine and polyamine biosynthesis (ARG + POLYAMINE-SYN, *r*^2^ = 0.46), polyamine biosynthesis I (POLYAMSYN-PWY, *r*^2^ = 0.45) and polyamine biosynthesis II (POLYAMINSYN3-PWY, *r*^2^ = 0.44). Pathways exhibited a stronger correlation to forearm samples, with an average *r*^2^ = 0.14 for pathways having MDS1 scores less than 0, aligning with the observed higher Shannon entropy in the forearm samples as compared to the face. In contrast, the average *r*^2^ = 0.07 for pathways having MDS1 scores greater than 0. Of the 1,411 species in the full 16S feature table, *Streptococcus* spp. had the highest number of ASVs which were mapped to either of these four pathways (by parsing the PICRUSt2 stratified output abundance table), suggesting a potential functional contribution of *Streptococcus* spp. within these metabolic pathways.

### Skin lipid profile differs between body sites

Total FFA, ceramides, cholesterol and cholesterol sulfate in the skin surface were quantified and the percentages of each ceramides subtypes were measured by SFC-MS/MS (Table 1). The PCoA plot of the lipid profiles using a Euclidean distance matrix showed significant separation between the body locations (Figure 2C). Females and males have different lipids profiles in the face samples (pairwise adonis *q*-value = 0.01), not in the forearms (pairwise adonis *q*-value = 0.09). No significant differences were observed in age and skin type groups (data not shown). Total FFA, cholesterol sulfate, sphingosine (AS and NS) are more abundant in the face, while Dihydro-/6-Hydroxy/phyto-Ceramides (NH, NP, AH, AP) are more abundant in the forearm (Supplementary Figure S5).

### Correlations between skin microbiome and lipid profiles

**Figure 4:**
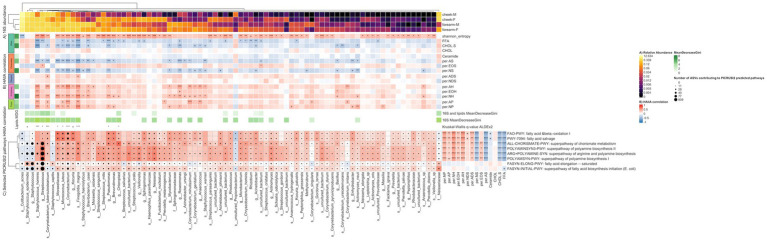
Heatmap illustrating the relationship between 16S species, representative predictive metabolic pathways and lipids. Columns represent 84 species discriminating between location and gender. The discriminating species were selected as the union of (i) species having at least one significant (*q*-value <= 0.05) HAllA correlation to lipids or Shannon entropy (ii) species selected using the Boruta feature selection algorithm for the Random Forest model using only species as input, and (iii) species selected using the Boruta feature selection algorithm for the Random Forest model using species and lipids as input. The MeanDecreaseGini score represents feature importance in the Random Forest classification model. Benjamini-Hochberg *q*-values (HAllA correlations) and Kruskal–Wallis *q*-values (ALDEx2 differential abundance) represent ‘***’ 0–0.001, ‘**’ 0.001–0.01, ‘*’ 0.01–0.05, ‘+’ 0.05–0.1. **(A)** Relative abundance of 16S species per location-gender group **(B)** HAllA correlation of 16S species to lipids. Lipids are grouped by their Sphingoid Base (Ceramides, Dihydro-ceramides, 6-Hydroxy-ceramides, Phyto-ceramides) or Other. **(C)** Rows represent 8 PICRUSt2 predicted pathways, amino-acid and lipid-related, discriminating between location or gender (ANCOM-BC *q*-value <= 0.05). Cells are colored by HAllA correlation between 16S species and predicted pathways. Black circles within cells are scaled by the number of ASVs contributing to PICRUSt2 predicted pathways. HAllA correlation between discriminant predicted pathways and lipids shown to the right. F, female; M, male.

To explore the relationship of the skin microbial composition and lipids profiles, HAllA analysis was performed. Eighty-two species clusters were identified to have significant correlation with Shannon diversity and/or lipids (Supplementary Table S2). The most significant bacteria cluster including *S. hominis, Corynebacterium* sp.*, Corynebacterium tuberculostearicum, Finegoldia magna* had positive correlation with Shannon diversity, dihydro- (ADS)/6-hydroxy (AH, NH)/phyto-cCeramides (AP, NP), and negative correlation with FFA, cholesterol sulfate, sphingosine based ceramides (AS, NS). A few other bacteria, for instance, *Pseudomonas* sp., *Moraxellaceae* sp. and *Roseomonas* sp. and *Brevibacterium casei* had similar correlation patterns as Cluster 1. In contrast, *C. acnes* tended to have inverse correlation patterns as compared to the clusters described above. They are positively correlated with FFA, cholesterol sulfate, sphingosine based ceramides, and negatively correlated with dihydro−/6-hydroxy/phyto-ceramides. The relationship among Shannon entropy, representative bacteria clusters and lipids was illustrated in Figure 4.

To further explore the potential mechanism of the correlation between the species and lipids, HAIIA analysis was run using the predictive metabolic pathways generated by PICRUSt2 and lipids. Hundred and forty-seven pathway clusters of species were identified to have significant correlation with lipids (Supplementary Table S3). The most representative pathway cluster is composed of 9 lipids and 226 pathways, which are involved in amino acids metabolism, carbohydrates degradation, and aromatic compounds metabolism. This cluster is positively correlated with ADS, NP, AP, AH, NH, while negatively correlated with FFA, cholesterol sulfate, AS, NS. Many of the pathways in this cluster have positive correlation to *S. hominis*, *Corynebacterium tuberculostearicum and Corynebacterium* sp. (Figure 4); all species are more abundant on the forearm.

Additionally, we performed HAIIA analysis to explore the correlation between bacteria species and predictive metabolic pathways, especially lipid related pathways. We observed lipid related pathways that are significantly correlated with certain bacteria (Supplementary Table S4). For instance, *S. hominis, C. tuberculostearicum, Micrococcus luteus, Finegoldia magna*, and *Moraxellaceae* sp. are more location specific and had significant positive correlations with fatty acid degradation pathway (FAO-PWY: fatty acid β-oxidation I (generic); PWY-7094: fatty acid salvage). *S. epidermidis* and family Neisseriaceae had significant correlations (q-value <= 0.05) with fatty acid synthesis pathways including super pathway of fatty acid biosynthesis initiation (FASYN-INITIAL-PWY) and fatty acid elongation -- saturated pathways (FASYN-ELONG-PWY). The correlations of representative predicted metabolic pathways and bacteria/lipids are illustrated in Figure 4. The correlations of all predicted pathways having a significant q-value and bacteria/lipids are illustrated in Supplementary Figure S6.

## Discussion

Skin microbial composition varies by the body locations and is highly influenced by age, gender and skin types etc. (Grice et al., 2008; Ross et al., 2017; Li et al., 2019; Boxberger et al., 2021). Our findings are consistent with other studies showing significant differences in skin microbial composition between body locations and gender. The female forearm has the highest microbial diversity (Figure 3) and certain bacteria were identified as either location specific, such as *S. hominis, M. luteus, C. tuberculostearicum, F. magna* and *Moraxellaceae* sp.; or gender specific, e.g., *Streptococcus* sp. and *S. epidermidis* (Supplementary Figure S4). More interestingly we found that predicted bacteria metabolic pathway profiles differed by body location and gender as well. Predicted pathways involved in arginine, polyamine, and chorismate biosynthesis and fatty acid degradation are more abundant in the female forearm, and sulfur oxidation and fatty acid biosynthesis pathways are more abundant in male face. The differences in skin microbial composition and metabolism might be due to the difference in sweat or sebum production, cosmetics application, skin pH, thickness, or hormone production which may favor the growth or activity of specific bacteria in a certain body location or gender (Grice and Segre, 2011; Ross et al., 2017). The bacterial species statistically enriched according to body location and gender show phylogenetic conservation of genes involved in specific metabolic pathways suggests that they may be involved in these metabolisms in the skin. For instance, *S. hominis, M. luteus, C. tuberculostearicum, F. magna* and *Moraxellaceae* sp. may be involved in arginine/polyamine biosynthesis, chorismate biosynthesis and fatty acid degradation, while *Streptococcus* sp. and *S. epidermidis* may be involved in sulfur oxidation and fatty acid biosynthesis metabolism. Further investigation into the actual genomic content of these organisms, and their expression *in situ*, will be necessary to establish these relationships.

The composition and distribution of skin lipids also vary by the body sites (Ludovici et al., 2018). Different ceramides subclasses are also distributed differently. In line with the previous study showing the relative abundance of NS is higher in forehead than arm in healthy and atopic skin, and NH is higher in arm than forehead (Emmert et al., 2021), we observed that total FFA, cholesterol sulfate, sphingosine (AS and NS) are more abundant in the face, while dihydro−/6-hydroxy/phyto-ceramides (NH, NP, AH, AP) are more abundant in the forearm. It may be due to the different sebaceous gland density and secretion in different body sites (Ludovici et al., 2018).

Age and skin type also impact the skin microbiome composition and/or lipid profiles (Rogers et al., 1996; Grice et al., 2008). However, in this cohort, we did not observe significant differences in skin microbiome or lipids profiles between skin types and age groups. This study relied on self-reported skin type, so the lack of significance may be due to incorrect assessments. The low number of participants in each age group would also reduce our ability to identify significant differences.

The novel discovery from this study is we observed a unique pattern of the interaction between skin bacteria and lipids. For instance, *S. hominis, Staphylococcus* sp.*, C. tuberculostearicum, F. magna* are positively co-correlated with Shannon diversity and skin lipids especially the essential ceramides (AP, NP), indicating a potential functional ecotype where these bacteria are replying on and/or involving in production of a particular skin ceramides profile. *S. hominis* is the second most frequently isolated coagulase-negative staphylococci (CoNS) from healthy skin (Kloos and Schleifer, 1975; Becker et al., 2014). Researchers have shown that *S. hominis* is a protective CoNS preventing pathogenic *S. aureus* from colonizing or infecting the skin (Severn et al., 2022). The correlation between *S. hominis* and skin ceramides suggested that *S. hominis* could be a beneficial bacteria to help build the skin ceramides, similar to its closely related species *S. epidermidis*, which has been shown to produce ceramides to maintain skin barrier (Zheng et al., 2022). Therefore, we hypothesize that *S. hominis* and other bacteria in the same clusters could be beneficial to enhance skin barrier function.

We further conducted PICRUSt2 analysis to predict the functional metabolic pathways of the skin microbiome using characterized 16S rRNA gene sequences and correlated the pathways to bacteria and the lipids. The correlation between skin lipids, skin bacteria and representative predicted metabolic pathways is illustrated in Figure 4. We did not observe any ceramides synthesis pathways that are correlated with *S. hominis, C. tuberculostearicum, Micrococcus luteus, Finegoldia magna, Moraxellaceae* sp. In contrast, these bacteria had significant positive correlations with fatty acid oxidation and salvage pathways, indicating that these bacteria may be also involved in long chain fatty acid degradation metabolism. While *S. epidermidis* and bacteria from family Neisseriaceae had significant correlations with fatty acid elongation and initiation synthesis, indicating that these bacteria may be involved in fatty acid biosynthesis.. However, the hypothesis was generated based on computational correlation analysis. Future *in situ* studies are needed to further explore the mechanism of the relationship between the bacteria and skin lipids.

In summary, we investigated the impact of body location and gender on skin microbial composition, functional metabolic pathway profiles and skin lipids profiles, and revealed unique patterns of interactions between skin bacteria and lipids. This study provides valuable insights on the relationship of skin microbiome and lipids, and gains a deeper understanding of how skin microbiome shapes and is being shaped by skin lipids.

## Data availability statement

The 16S rRNA sequencing data is publicly available at https://www.ncbi.nlm.nih.gov/bioproject/?term=PRJNA1073917.

## Ethics statement

The studies involving humans were approved by Concordia Institutional Review Board (IRB) 7 East Frederick Place Ceadr Knolls, New Jersey 07927 Telephone: (973)734-0734. The studies were conducted in accordance with the local legislation and institutional requirements. The participants provided their written informed consent to participate in this study.

## Author contributions

ML: Conceptualization, Data curation, Formal analysis, Funding acquisition, Investigation, Project administration, Writing – original draft, Writing – review & editing. EK: Data curation, Formal analysis, Methodology, Visualization, Writing – original draft, Writing – review & editing. JM: Funding acquisition, Supervision, Writing – review & editing. JN: Data curation, Formal analysis, Methodology, Writing – review & editing. JS: Formal analysis, Methodology, Writing – review & editing. JW: Conceptualization, Funding acquisition, Writing – review & editing.
