# Supplementary material for: Microbiome and lipidomic analysis reveal the interplay between skin bacteria and lipids in a cohort study
Source: Front Microbiol. 2024 Apr 11;15:1383656. doi: 10.3389/fmicb.2024.1383656 (PMC11043602; doi:10.3389/fmicb.2024.1383656)
Supplement: Supplementary file 4 [file Data_Sheet_1.docx]

**Supplementary Figures and Tables**

Supplementary Table 1. 271 metabolic pathways identified by PICRUSt2 (q-value <= 0.05) showing differential abundance between location and gender.

Supplementary Table 2. 84 bacteria clusters were identified to have significant correlation with Shannon diversity and/or lipids by HAIIA.

Supplementary Table 3. 147 pathway clusters were identified to have significant correlation with lipids.

Supplementary Figure 1. Alpha diversity of the skin microbiome in different (A) age groups and (B) skin types characterized by 16S rRNA sequencing.

Supplementary Figure 2. Relative abundance of select bacterial species identified as significantly differentially abundant between body location and gender identified by 16S rRNA gene sequencing. ALDEx2 Wilcoxon Rank Sum Test q-values represent ‘***’ 0-0.001, ‘**’ 0.001-0.01, ‘*’ 0.01-0.05, ‘+’ 0.05-0.1.

Supplementary Figure 3. Random Forest Out-of-Bag error rates for 16S rRNA sequencing data and Lipids profile.

Supplementary Figure 4. (A) MDS plot displaying sample proximities (scaled to [-1, 1]) with 95% confidence ellipses, based on Random Forest model for 16S and Lipids with gender and location as response. PICRUSt2 pathways (ANCOM-BC q-value <= 0.05) fitted to MDS using envfit. Size of points for features (16S and lipids) scaled based on Gini score (B) Number of ASVs contributing to selected pathways, stratified by species (PICRUSt2 output)

Supplementary Figure 5. Discriminating skin lipids between body location and gender identified by lipidomic analysis. ADS: dihydroceramide alpha-hydroxy fatty acids , AH: 6-hydroxyceramide alpha-hydroxy fatty acids, AP: phytoceramide alpha-hydroxy fatty acids, AS: ceramide alpha-hydroxy fatty acids, EOH: 6-hydroxyceramide esterified omega fatty acids, EOS: ceramide esterified omega fatty acids, NDS: dihydroceramide saturated fatty acids, NH: 6-hydroxyceramide saturated fatty acids, NS: ceramide saturated fatty acids, NP: phytoceramide saturated fatty acids.

Supplementary Figure 6. Heatmap illustrating the relationship between 16S species, 271 predictive metabolic pathways (having q-value <= 0.05 in ANCOM-BC analysis for discriminating between location or gender) and lipids. Columns represent 84 species discriminating between location and gender. The discriminating species were selected as the union of (i) species having at least one significant (q-value <= 0.05) HAllA correlation to lipids or Shannon entropy (ii) species selected using the Boruta feature selection algorithm for the Random Forest model using only species as input, and (iii) species selected using the Boruta feature selection algorithm for the Random Forest model using species and lipids as input. The MeanDecreaseGini score represents feature importance in the Random Forest classification model. Benjamini-Hochberg q-values (HAllA correlations) and Kruskal-Wallis q-values (ALDEx2 differential abundance) represent ‘***’ 0-0.001, ‘**’ 0.001-0.01, ‘*’ 0.01-0.05, ‘+’ 0.05-0.1. (A) Relative abundance of 16S species per location-gender group (B) HAllA correlation of 16S species to lipids. Lipids are grouped by their Sphingoid Base (Ceramides, Dihydro-ceramides, 6-Hydroxy-ceramides, Phyto-ceramides) or Other. (C) Rows represent 271 PICRUSt2 predicted pathways discriminating between location or gender (ANCOM-BC q-value <= 0.05). Cells are colored by HAllA correlation between 16S species and predicted pathways. Black circles within cells are scaled by the number of ASVs contributing to PICRUSt2 predicted pathways. HAllA correlation between discriminant predicted pathways and lipids shown to the right. F: female, M:male.
