# Supplementary figures and images for: Microbiome and lipidomic analysis reveal the interplay between skin bacteria and lipids in a cohort study
Source: Front Microbiol. 2024 Apr 11;15:1383656. doi: 10.3389/fmicb.2024.1383656 (PMC11043602; doi:10.3389/fmicb.2024.1383656)

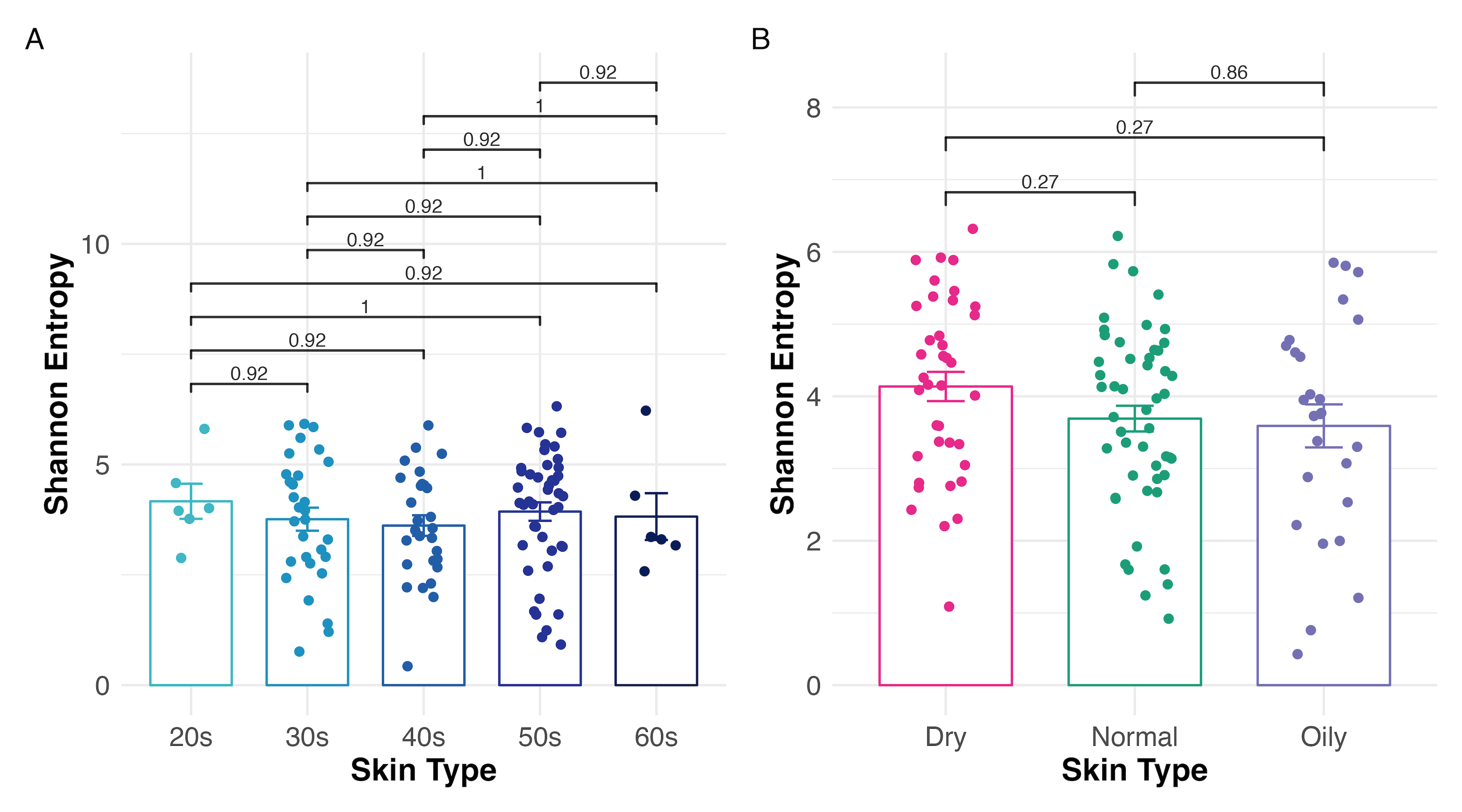

Supplement: Supplementary file 5 [file Image_1.TIFF]

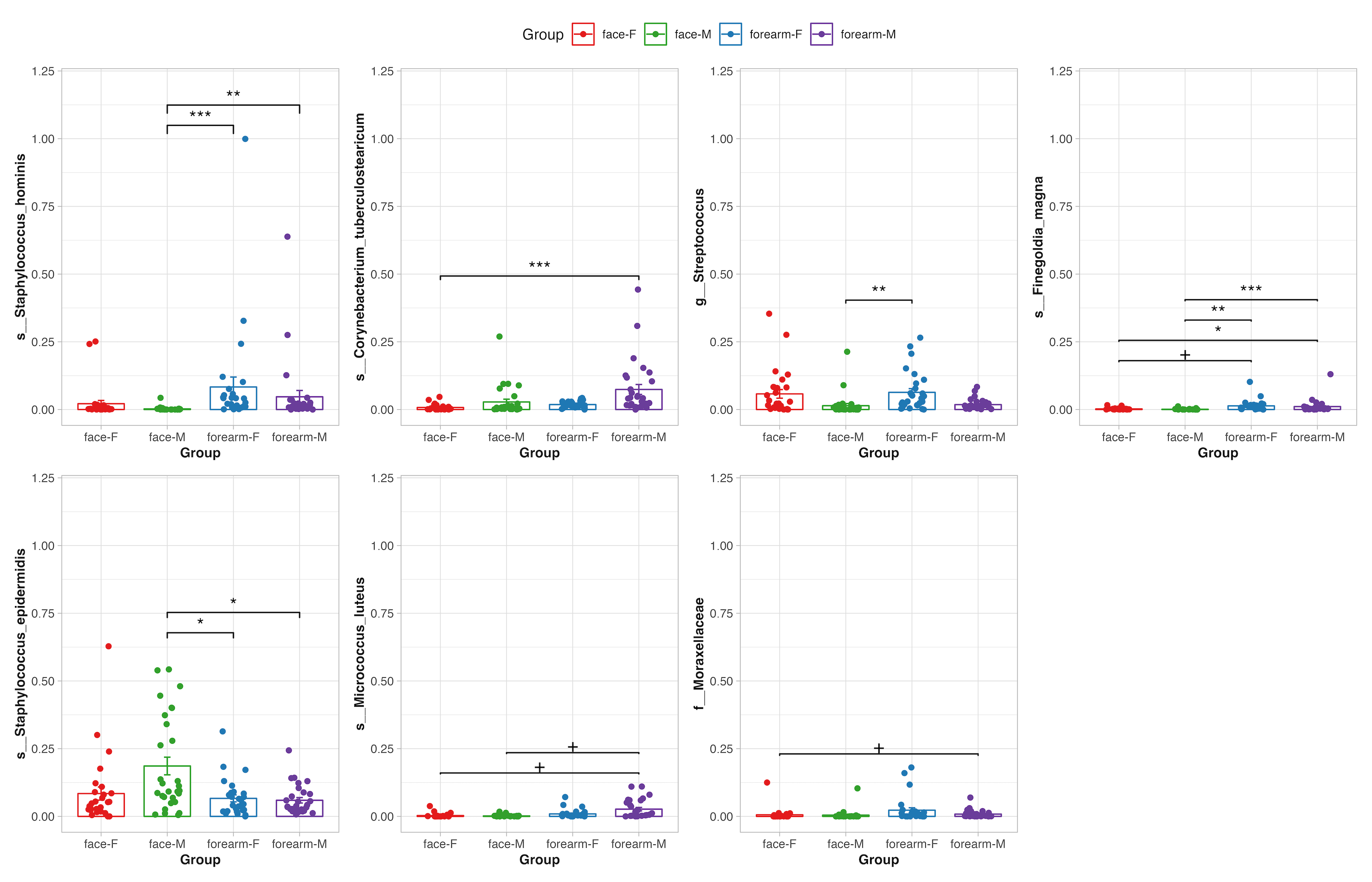

Supplement: Supplementary file 6 [file Image_2.TIFF]

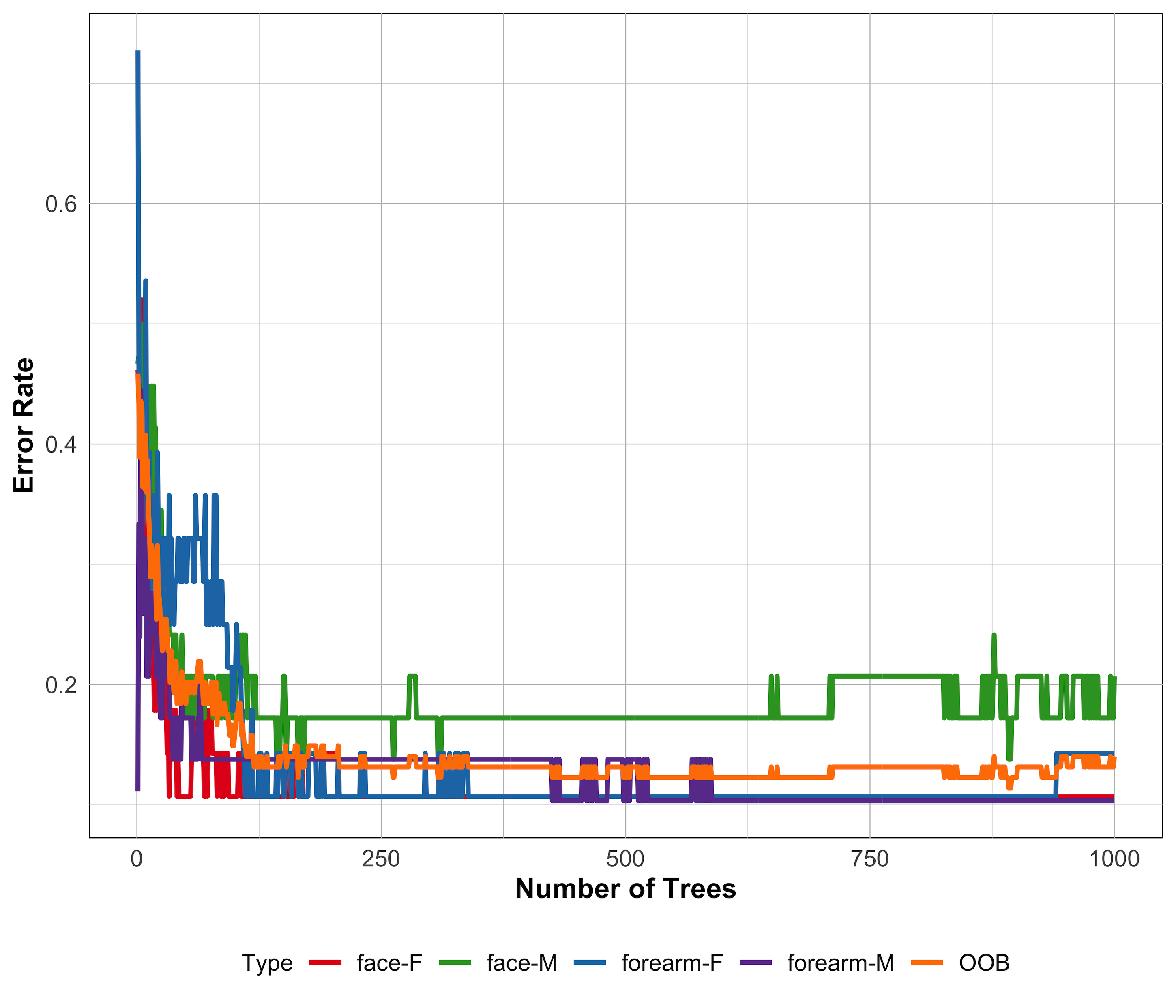

Supplement: Supplementary file 7 [file Image_3.TIFF]

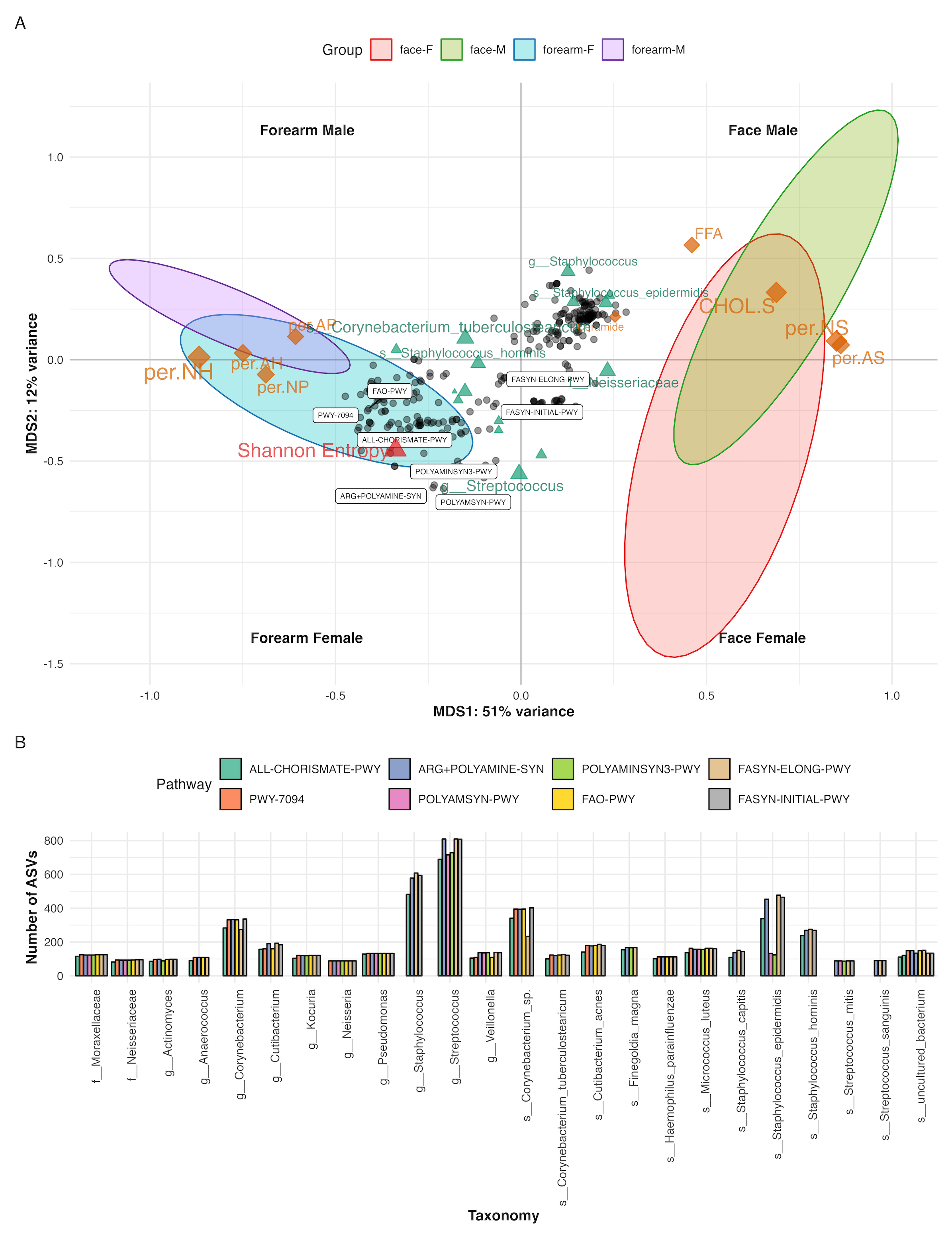

Supplement: Supplementary file 8 [file Image_4.TIFF]

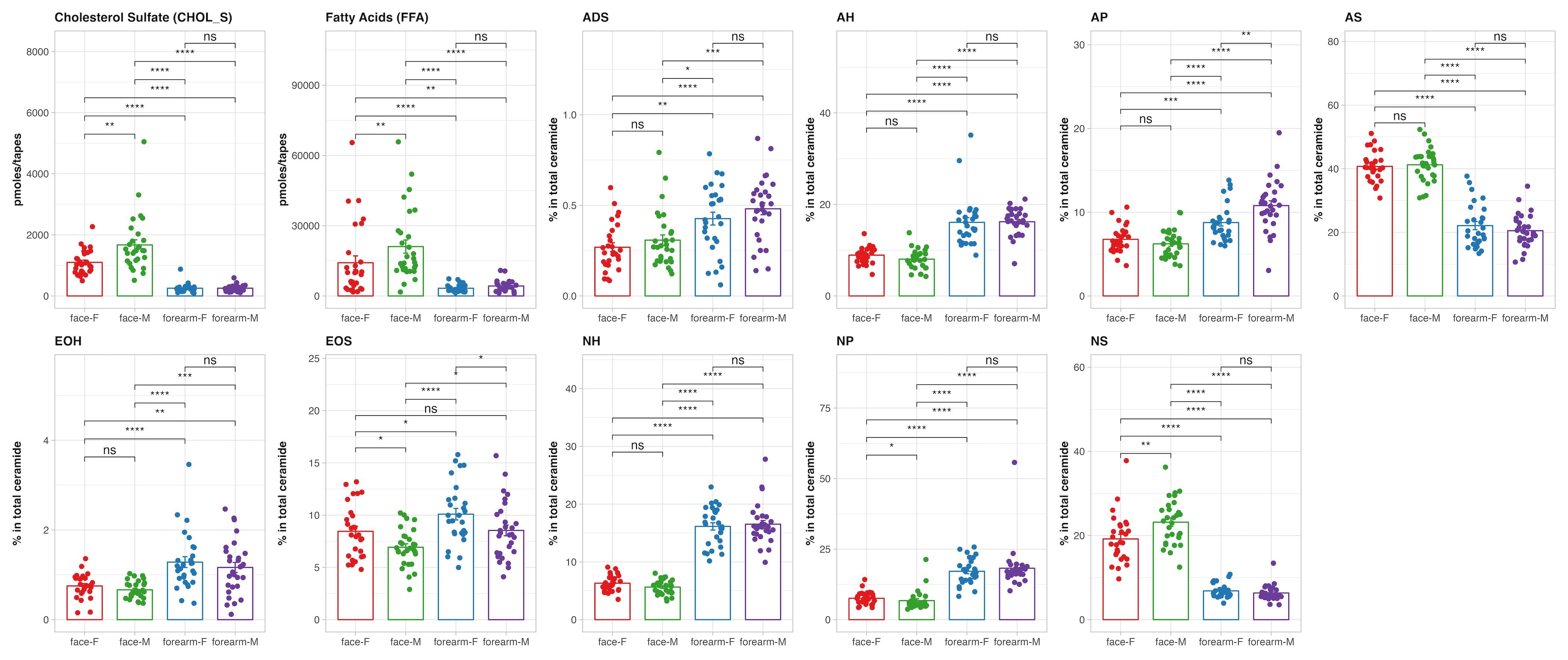

Supplement: Supplementary file 9 [file Image_5.TIFF]

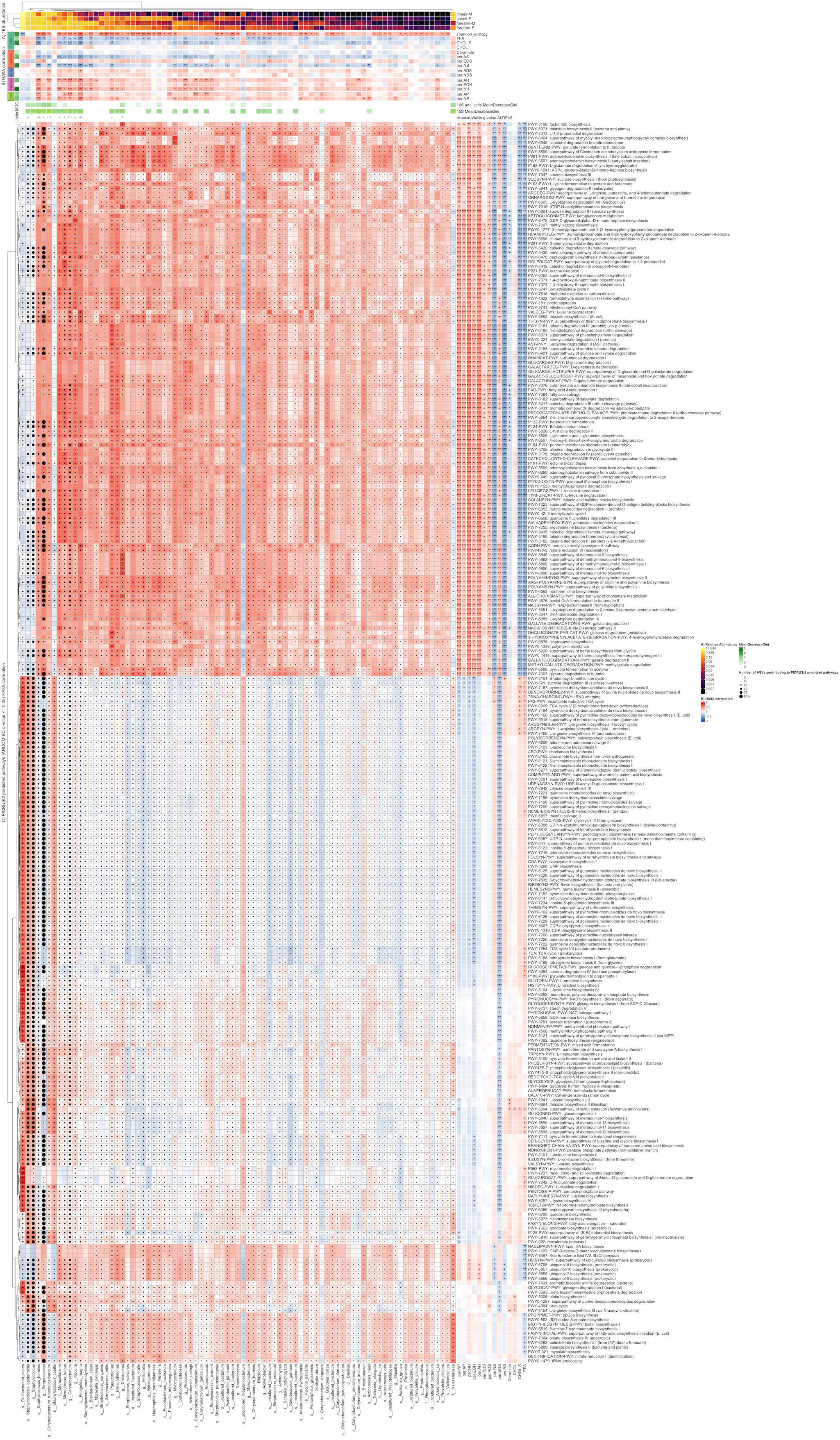

Supplement: Supplementary file 10 [file Image_6.TIFF]
